# Response of woody vegetation to bush thinning on freehold farmlands in north-central Namibia

**DOI:** 10.1038/s41598-022-26639-4

**Published:** 2023-01-06

**Authors:** Matti T. Nghikembua, Laurie L. Marker, Bruce Brewer, Arvo Leinonen, Lauri Mehtätalo, Mark Appiah, Ari Pappinen

**Affiliations:** 1 Biomass Technology Centre, Cheetah Conservation Fund, Elandsvreugde 367, Otjiwarongo, 12001 Namibia; 2 School of Forest Sciences, University of Eastern Finland, Joensuu Campus, Yliopistokatu 7, Joensuu, 80101 North Karelia, Finland; 3 Bioenergy Consult Arvo Leinonen, Huikkolantie 21, Vaajakoski, 40800 Jyväskylä, Finland; 4 Natural Resources Institute Finland (LUKE), Yliopistokatu 6, 80101 Joensuu, Finland; 5 CSIR-Forestry Research Institute of Ghana (CSIR-FORIG), P. O. Box UP 63 KNUST, Kumasi, Ghana; 6 Faculty of Natural Science and Environmental Management, Department of Natural Resources Management, CSIR College of Science and Technology (CCST), P.O. Box M32, Accra, Ghana

**Keywords:** Ecology, Plant sciences, Environmental sciences

## Abstract

Bush encroachment affects much of the Namibian woodland landscape, causing significant loss of open savannah habitat and farm profits. Thinning of the trees/shrubs is recommended; however, research is required to identify the overall efficacy and effects of this method on the woodland habitat. We aimed to examine the effect of the thinning strategy applied on the vegetation structure of encroaching tree/shrub species, as well as the sighting lines of the habitat. Vegetation surveys were done on three freehold farms in north-central Namibia. The study utilised a combination of a blocked and split-plot study design: each block consisted of a pair of thinned and non-thinned plots with multiple subplots. Thinned plots had been manually thinned, with a post-thinning age of three years or more. Results revealed that tree/shrub abundance differed between species; thinned areas had the least abundance and overall species-treatment interactions were significant. Thinning caused a significant reduction in overall tree/shrub densities, settling within the recommended range for the area. Thinning also significantly reduced the average tree/shrub height, canopy area, medium-sized trees/shrubs, and increased sighting lines. This confirms a bush encroachment mitigation strategy that favours grass cover, and wildlife that rely on longer sighting lines for safety or when hunting.

## Introduction

Namibian farmland has undergone significant ecological changes as a result of anthropogenic influences, resulting in an increase in the density and biomass of native woody species, a phenomenon known as bush encroachment^1–3^. Approximately 45 million hectares (ha) of the territory has been affected, causing significant declines in the grazing carrying capacity, beef production, suitable habitats for some grazing ungulates, and predator numbers (e.g., cheetah *Acinonyx jubatus*)^1,3–5^. This phenomenon also has potential to show varied impacts to the local climates according to geographic region. For example, in the humid northern temperate regions (southwest of North America and center of Central Asia), shrub encroachment in grasslands lowered daytime land surface temperatures and albedo, but increased evapotranspiration that is typically associated with heat loss^6,7^. In contrast, higher land surface temperatures were experienced at nighttime, due to the vegetation shading effects. In the arid and semi-arid northern temperate regions, shrub encroachment led to the increase in daytime and nighttime land surface temperatures, reduction in leaf area index and evapotranspiration, however, albedo was increased^7^. These effects were attributed to higher fraction of bare soil patches in encroached areas that experiences heat fluxes during the day and greater outgoing longwave radiation at night. Also, the reduced evapotranspiration experienced accounted for less heat loss and cooling effect, consequentially causing warming. Shrub encroachment has also been observed to correspond positively to the increase in land surface temperatures and extended growing seasons in China’s temperate grasslands region^8^. Effects of this phenomenon towards the local land surface temperatures are unknown on Namibian rangelands. However, bush encroachment may have some warming and cooling effect due to higher evapotranspiration, vegetation shading effects and presence of bare soil patches that could contribute to the absorption and reflection of heat energy.

To counteract the negative effects of this phenomenon, restoration thinning selectively removes the overabundant trees/shrubs and is recommended since it minimises negative impacts on the vegetation, habitats or biodiversity^2^. Harvesting of trees/shrubs has potential to alter the structure or dynamics of their population as woody densities and biomass are reduced, and compositions may change due to some species with certain growth characteristics being preferred or avoided. Also, the reduction of densities releases competition for resources (e.g., water, nutrients, sunlight), causing the retained individuals to increase their growth rates, reproduction, and enhancing sapling survival^9,10^. Trees/shrubs also play a key role in carbon sequestration and the nutrient cycle in savannah ecosystems: the sub-habitats underneath canopies or near dead trees contain substantial concentrations of soil elements, such as nitrogen (N), organic matter (OM), calcium (Ca), phosphorus (P), potassium (K), and carbon (C)^11–15^. Also, trees/shrubs increase the soil microbial biomass that is important to nutrient cycling, including the mineralization of plant, animal and organic carbon, and leguminous tree/shrub species are also N fixers^11,16–18^. Severe or uncontrolled bush harvesting may result in the disruption of all these crucial roles played by the vegetation in the savannah environment. However, if done in moderation, it may not disrupt the stability of the ecosystem^19,20^. Protective measures with regard to biodiversity and the vegetation are crucial as they provide ecological services, which include nutrient cycling, erosion control, water cycling, carbon sequestration, forage for animals, breeding sites, cover against predators or adverse weather conditions, construction materials, and a source of energy^2,12–15,17,21^.

The farmland matrix serves as an important habitat for livestock farming and biodiversity conservation. As an example, the majority (> 80%) of cheetah and numerous protected and economically important large ungulates, including eland (*Tragelaphus oryx*), oryx (*Oryx gazella*), greater kudu (*Tragelaphus strepsiceros*) and red hartebeest (*Alcelaphus buselaphus* ssp. *caama*), reside on freehold farmlands^22–24^. The restoration of bush-encroached habitat to open shrubland would increase grazing carrying capacity, and consequentially, would lead to greater farmland profits and boost prey populations, thereby reducing the likelihood for human-wildlife conflict^1,25^. Hence, for this reason, inventory and monitoring of restoration thinning habitats are increasingly important aspects for sustainable livestock management and biodiversity conservation, and should lead to appropriate management actions.

Absolute population estimates (expressed per unit area) are commonly applied to describe the abundance of species in a specific habitat, evaluate management impacts on species and habitat suitability for wildlife and associations of plant species^21,26–30^. Density (expressed as the number of individuals per unit area) is one such estimate. Attributes, such as height, canopy diameter or stem diameter often accompany density estimates to quantify biomass volumes and vegetation structure^9,31–33^. Biomass (wet, dry weight, organic carbon content) contained in trees/shrubs is useful in understanding the trophic structure, carbon sequestration potential, availability of biomass for offtake, or dominance of species in a particular area^31,34–37^. Such estimates are obtained either directly through destructive sampling, or indirectly using allometric equations that relate oven dry weight with tree/shrub attributes (e.g. height, stem diameter, canopy diameter or spatial canopy volume)^31,38^.

Indirect acquisition of estimates is efficient due to lower required labour intensity and the time required to obtain samples directly. However, there is a lack of verified models for some species, since current models cannot be applied universally due to differences between species or variations in environmental conditions that could cause over/under estimation of the biomass^35,36,39^. However, a number of allometric equations, especially suitable for the small, multi-stemmed bush-encroaching species found in semi-arid landscapes in southern Africa, have been published^31,40^. Hence, use of these allometric models may be appropriate to estimate available bush biomass in our study area since they were developed for almost similar species and region^39,41^.

The need to restore rangeland productivity and wildlife habitats by thinning the overabundant biomass was the rationale for Cheetah Conservation Fund (CCF) to establish the CCF Bush Project in 2001^42,43^. To date, a number of studies have reported the impacts of this project on soil fertility, vegetation regeneration, and the response of wildlife^17,28,44,45^. Our study differs from previous research in that it examines (a) the effects of treatment (thinned *vs* non-thinned) on the abundance (density, biomass) and structure (height, canopy area, stem size) of bush-encroaching species, and (b) evaluates habitat sighting lines, to gain insight into how effective this method has been in reducing bush encroachment on the north-central freehold farmlands of Namibia.

We focused the research on three questions: (1) how do thinned and non-thinned areas differ in vegetation structure (i.e., height, canopy area, stem size distribution) and abundance (density, woody biomass quantity), (2) in what ways has selective thinning influenced tree/shrub density (expressed as tree equivalents (TE) ha^−1^) recommended for the area, and (3) in what ways has selective thinning affected sighting lines, a key habitat feature associated with cheetah hunting success. We hypothesized that thinning would reduce the abundance of encroaching species, that the magnitude of the impacts at thinned sites would differ between tree/shrub types, and that thinning would significantly modify the vegetation structure and restore sighting lines for improved detection. This study provides baseline data with regard to the vegetation structure in both encroached and previously thinned areas, which will allow evaluation of the efficacy of restoration thinning and assist with the recommendation of appropriate management actions for the affected areas.

## Material and methods

### Study area

**Figure 1:**
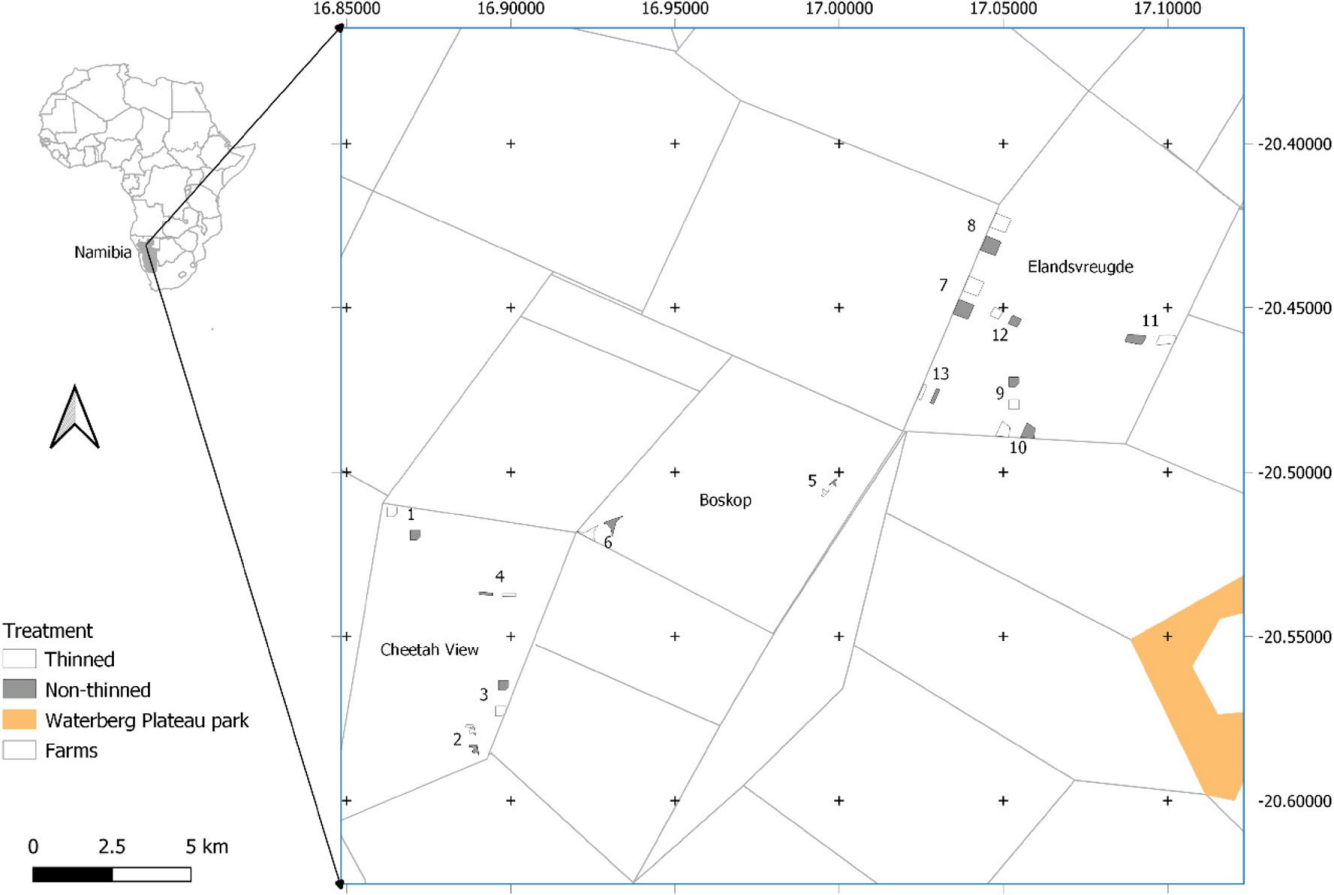
Location of the study area within the north-central farmland of Namibia. Grey squares indicate non-thinned and open squares indicate thinned plots. Block pairs are numbered 1–13. Map created with QGIS Geographic Information System software, version 3.18.3-Zürich, https://www.qgis.org.

The study area consisted of three freehold farms; Cheetah View (#317), Boskop (#324) and Elandsvreugde (#367), in north-central Namibia (farm size: 5046–7300 ha); coordinates: 20.42407° S–20.58440° S, 16.86337° E–17.09950° E (Fig. 1). Elevation is approximately 1580 m; the topography of the farms is generally flat and there are no perennial rivers. The farms were developed for livestock and are surrounded by cattle proof fences (~ 1.5 m high) that allow wildlife movement. Semi-permanent waterpoints provide year-round access to water. The region experiences a semi-arid climate with three main seasons: hot-wet (January–April), cold-dry (May–August) and hot-dry (September–December). Mean annual rainfall is 401 mm (± 257.4), which is concentrated mainly between January–April. Mean annual temperature is 19.2 °C (± 2.4), and mean daily maxima is 22.7 °C (± 0.7) in January and 13.4 °C (± 0.7) in July^46^.

The predominant soil types are Eutric Regosols and Chromic Cambisols^44,47^. Soil texture types consist of sandy loam and loamy sand. The vegetation is broadly classified as thornbush shrubland, dominated by *Dichrostachys cinerea* (L.) Wight & Arn*, Senegalia mellifera* (Vahl) Seigler & Ebinger*, Vachellia reficiens (*Wawra & Peyr.) Kyal. & Boatwr.*, Terminalia sericea* Burch. ex DC.*, Terminalia prunioides M.A.*Lawson*, Combretum imberbe* Wawra*, Grewia flava* DC. and *Grewia flavescens* Juss. species^47,48^.

### Species of interest

Observations focused on five thornbush woody species known to cause encroachment in the area: sickle bush (*D. cinerea* subspecies spp. *africana*), black-thorn acacia (*S. mellifera* subspp. *detinens*), blade thorn (*Senegalia cinerea* (Schinz) Kyal. & Boatwr., red umbrella thorn (*V. reficiens*), and umbrella thorn (*Vachellia tortilis* (Burch.) Kyal. & Boatwr. spp. *heteracantha*). These native trees/shrubs are widespread in the region. Distinguishing features of these species include sharp thorns and spines (straight and hooked), as well as fine compound and bipinnate leaves. These species have economic value since local communities rely on their raw material for fuelwood, charcoal production, as handles for axes/picks, fencing poles, construction materials, as forage for livestock and game, and as an ingredient in the production of animal feeds and traditional medicine^49–51^.

All five encroaching species were manually thinned using handheld tools (axes and machetes) to reduce their densities by approximately 50%. Thinning of trees/shrubs was random and included all height classes to retain a heterogeneous mix of tree/shrub heights. Smaller tree/shrubs (≤ 4 m. height, ≤ 18 cm stem diameter) were the most selectively thinned (40–60%) due to their higher abundance in the study area. Larger trees/shrubs with ≥ 18 cm stem diameters were avoided. Some vegetation clumps were left undisturbed to provide cover, browse, shelter or breeding sites for wildlife.

### Study design and data collection

A combination of a blocked and split-plot study design was used in this study. This included 13 blocks (also called plot pairs), each with two plots with a mean (± standard deviation) size of 12.62 (± 8.43) ha, one for each treatment (Fig. 1). For the collection of vegetation data, 4–20 circular subplots with a 6 m radius (area = 113.1 m^2^) were placed within each plot. A total of 295 subplots were surveyed: 49.5% (n = 149) were in the thinned and 50.5% (n = 146) in the non-thinned treatment area. The treated plots were established on areas that were manually thinned from 2002 to 2013 with a post-thinning age equal to 3 years or more in 2016, so that multiple seasons followed thinning. A single thinning cycle has been conducted, and tree/shrub densities were reduced by approximately 50%. Since 2005, aftercare treatment to prevent stump regrowth was carried out using Picloram and Tryclopyr active arboricide (Access*®*) on freshly cut stumps. Eleven previously thinned plots had been treated with Access® and two plots that had been thinned earlier were not treated. The control treatment plots were established on areas where thinning had not been conducted. These were areas near the thinning treatment, located at a mean distance of approximately 0.71 (± 0.17) km in the same habitat that was affected by bush encroachment, with similar tree/shrub species and management history^28,45^.

A systematic random plot sampling method was used to designate the vegetation subplots, which were located 100 m apart in a plot. Vegetation data was collected from April to August 2017. We quantified sighting lines at the subplot level by positioning an observer who crouched 0.65 m aboveground, and measured the initial distance at which objects (human) could not be detected by an observer^4,52^. The starting bearing (direction) was random, three other similar measurements were added sequentially at 90° from the previous bearing. We calibrated the measurements by allowing a second person (preferably wearing non-bright colors) to walk away from the observer until they could not be seen. A one-meter accuracy rangefinder (Bushnell Yardage Pro Scout 6 ×) was used to take the measurements.

In addition, all encroaching trees/shrubs present in each subplot were assessed as follows: (1) counts of individuals for all target species, (2) tree/shrub height using an extendable marked polyvinyl chloride (PVC) pipe (3 m length), a standard ruler (30 cm) and a measuring tape (30 m) (for tall trees/shrubs (i.e., > 3 m high), the PVC pipe was used to measure heights), (3) tree/shrub condition based on two appearance categories: alive or dead, (4) maximum canopy diameter taken with a 30 m measuring tape from two perpendicular directions (d1, d2), and (5) base (stem) diameter, recorded at 10–15 cm aboveground.

### Aboveground biomass quantification

Aboveground biomass of the encroaching species (live individuals) was estimated with the allometric regression equation developed for Namibian thornbush species^40^ that relate biomass with tree/shrub height and stem diameter or the combination of both as predictors: y = ax^b^, where y = dry woody biomass; a = intercept, x = independent variable/s (tree/shrub height, stem diameter) and b = slope. For each tree/shrub, a combined biomass value was calculated for wood sizes ≤ 2 cm (ideal for bush feed), as well as > 2 cm (wood) (Appendix A1).

### Data analysis

A generalized linear mixed-effects model (GLMM) via penalized quasi-likelihood (glmmPQL) (package MASS, Venables and Ripley, 2002) was used to estimate how the number of trees/shrubs per species and stem diameter class were influenced by treatment:

$$E({y}_{kji})=exp({{\varvec{x}}\boldsymbol{^{\prime}}}_{kji}{\varvec{\beta}} +{b}_{k}+{c}_{kj}+{d}_{kji},)$$

where E (y_*kji*_) = mean number of tree/shrub counts; ***x****´*_*kji*_***β*** includes the effects of fixed factors; tree/shrub species or stem classes, treatment, the tree/shrub species-treatment or stem class-treatment interactions, arboricide application and post-thinning age (expressed as time since thinning minus 7.2 years), where 7.2 is the mean time since thinning over all treated plots; and b_*k*_ + c_*kj*+_ d_*kji*_ includes the nested random effects for block (plot pair), plot and subplot, respectively. For the non-thinned treatment, the post-thinning age was set to zero. We included aftercare application with arboricide in the estimation, as 11 previously thinned plots were treated and two had not been treated. Hypothesis testing for the GLMM model coefficients were based on Wald χ^2^ tests of the fitted final model using R functions Anova and lht of package car (Fox and Weisberg, 2011).

Linear mixed-effects (LME) models (package nlme, Bates et al., 2021) were used to estimate the influence of treatment on the overall tree/shrub density (TE), canopy size, woody biomass availability and sightlines in four separate models of the form:

$${y}_{kji}={{\varvec{x}}\boldsymbol{^{\prime}}}_{kji}{\varvec{\beta}} +{b}_{k}+{c}_{kj }+{e}_{kji},$$

where y_*kji*_ = mean number of trees/shrubs (TE), canopy size, woody biomass, and sighting lines; ***x****´*_*kji*_***β*** includes the effects of treatment, post-thinning age and arboricide aftercare as fixed factors; and b_*k*_ + c_*kj*_ includes the nested random effects for block (plot pair) and plot, with *e*_*kji*_ the subplot-level residual error, respectively. Further, the LME model was also used to estimate how the vertical structure (height) of the areas was affected by treatment:

$${y}_{kjil}={{\varvec{x}}\boldsymbol{^{\prime}}}_{kjil}{\varvec{\beta}} +{b}_{k}+{c}_{kj}+ {d}_{kji}+{e}_{kjil},$$

where y_*kjil*_ = mean tree/shrub height of the subplot; ***x****´*_*kjil*_***β*** includes the effects of fixed factors; treatment, post-thinning age and aftercare application; and b_*k*_ + c_*kj*_ + d_*kji*_ includes the nested random effects for block (plot pair), plot and subplot, with *e*_*kjil*_ the tree-level residual error, respectively. The fit of all final models was evaluated graphically by exploring the trends and homoscedasticity of the Pearson and deviance residuals; normal q–q plots of random effects were used to evaluate the assumption of the normality of random effects^55^. For all LME models, the non-constant residual variance was modelled by applying weighting (variance Power) following the inspections of the residuals in the initially fitted models:

*var*(ϵ_*kji*_) = σ^2^|y_*kji*_ |^2δ^, for tree/shrub densities (TE), canopy area, woody biomass, and sightlines and *var*(ϵ_*kjil*_) = σ^2^|y_*kjil*_|^2δ^ for vertical structure (height), where y_*kji*_ and y_*kjil*_ indicates group-level predictions for the models. Conditional F-tests on the fixed effects were carried out to determine significant predictors. All analyses were carried out with R version 4.0.4^56^ at the *P* ≤ 0.05% significance level.

### Approval of bush thinning and field research

Permission to conduct field research on the farms was acquired from the landowner (Cheetah Conservation Fund); all harvest operations, ecological research and monitoring program were done in accordance with the laws of the Namibian Ministry of Environment Tourism and Forestry (METF) (research permits: 2151/2016, 2324/2017) and certified by the Forest Stewardship Council (FSC) for sustainable forestry (certificate: FSC-C004580).

## Results

### Number of tree/shrub observations and structural characteristics

A total of 4223 trees/shrubs from five species were observed of which most (92.5%) were alive and few (7.5%) were dead (Table 1). Live trees/shrubs were recorded in almost all subplots (97.97%), although none were recorded in six subplots (thinned = 3, non-thinned = 3). Dead trees/shrubs were recorded in 56.9% of subplots, with most of the observations in the non-thinned area (Table 1).

**Table 1:** Observations of trees/shrubs by species and measured structural characteristics in the thinned and non-thinned treatments.

Variable/common name	Thinned			Non-thinned			Total Abundance
	Abundance	Mean	SD	Abundance	Mean	SD	
**Overall (alive and dead)**							
Sickle bush	887	–	–	1034	–	–	1921
Black-thorn acacia	640	–	–	290	–	–	930
Blade thorn	318	–	–	340	–	–	658
Red umbrella thorn	115	–	–	388	–	–	503
Umbrella thorn	190	–	–	21	–	–	211
Total	2150	–	–	2073	–	–	4223
**Height (m)/ all live individuals**							
Sickle bush	848	0.94	0.74	841	1.52	0.91	1689
Black-thorn acacia	619	0.55	0.78	269	2.44	1.67	888
Blade thorn	310	1.35	0.99	329	1.96	1.16	639
Red umbrella thorn	111	1.48	1.49	370	2.66	1.49	481
Umbrella thorn	190	1.27	0.95	21	2.26	1.77	211
Total	2078	0.94	0.92	1830	1.97	1.32	3908
**Canopy diameter (m)**							
Sickle bush	797	0.93	0.81	667	1.53	1.08	1464
Black-thorn acacia	411	0.75	1.1	201	2.79	2.23	612
Blade thorn	284	1.51	1.27	268	2.1	1.3	552
Red umbrella thorn	101	1.94	1.97	247	2.84	2.2	348
Umbrella thorn	182	1.73	1.17	16	2.31	2.13	198
Total	1775	1.12	1.16	1399	2.06	1.68	3174
**Stem diameter (cm)**							
Sickle bush	755	2.44	2.25	748	2.72	3.23	1503
Black-thorn acacia	515	2.55	4.53	243	8.24	7.16	758
Blade thorn	291	3.89	3.9	308	4.6	4.7	599
Red umbrella thorn	107	5.54	7.19	362	7.98	6.9	469
Umbrella thorn	182	4.23	4.79	18	9.74	10.98	200
Total	1850	3.06	4.02	1679	5.07	5.8	3529
**Dead trees/shrubs**							
Sickle bush	39	–	–	193	–	–	232
Black-thorn acacia	21	–	–	21	–	–	42
Blade thorn	8	–	–	11	–	–	19
Red umbrella thorn	4	–	–	18	–	–	22
Umbrella thorn	–	–	–	–	–	–	
Total	72	–	–	243	–	–	315

The number of trees/shrubs counted are indicated as abundances; the height, canopy and stem diameters are expressed as averages (mean) with associated standard deviation (SD) values for each species in the non-thinned and thinned areas.

Tree/shrub height was measured amongst all 3908 live trees/shrubs (Table 1). Overall, red umbrella thorn and blade thorn were, on average, the tallest (> 1.5 m) species. Canopy diameter was measured for 81.2% (n = 3174) of the live trees/shrubs (Table 1). On average, canopy size per individual tree/shrub was greatest in red umbrella thorn, followed by black-thorn acacia, blade thorn and umbrella thorn (Table 1). Sickle bush had the smallest average canopy size of all species. Stem diameter was measured for 90.3% (n = 3529) of the live trees/shrubs (Table 1). On average, red umbrella thorn had the largest stem diameter (> 7 cm) per individual tree/shrub. Other species with large stem diameters (4–5 cm) were black-thorn acacia, blade thorn and umbrella thorn. Sickle bush had the smallest average stem diameter of all species (Table 1).

### Number of observed individuals and species-treatment interactions

**Figure 2:**
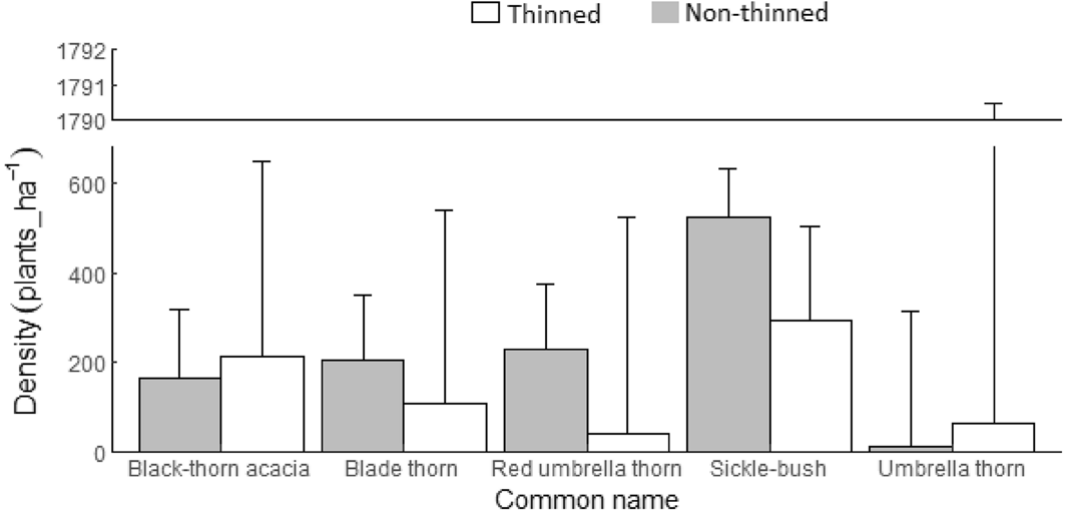
Expected number of tree/shrub densities (plants ha^−1^) in the thinned and non-thinned treatment areas estimated from the generalized linear mixed-effects model (GLMM) coefficients. Error bars indicate ± 95% confidence level of the variability in tree/shrub densities.

The baseline category (*β*_*0*_) of the GLMM model (Tables 2 and 3, Fig. 2) showed that the mean number of live sickle bush specimens counted in the non-thinned area was 5.91 individuals per subplot (equates to 522.74 plants ha^−1^). In the same treatment, all other encroacher species were less abundant than the baseline category (*β*_*0*_); mean counts of black-thorn acacia, blade thorn, red umbrella thorn, and umbrella thorn were 68.1%, 61%, 56.1%, and 97.5% different, respectively, from the live sickle bush count. The overall number of individuals counted in the non-thinned area was 12.85 (1136 plants ha^−1^).

**Table 2:** Generalized linear mixed-effects model (GLMM) showing the effects of fixed factors (treatment, post-thinning age, and arboricide aftercare) on the abundance of encroaching trees/shrubs.

Variable	Parameter (β)	Value	SE	Exp(β)
**Fixed effects**				
(Intercept)	*β*_0_	1.777	0.103	5.912
Black-thorn acacia	*β*_1_	− 1.142	0.166	0.319
Blade thorn	*β*_2_	− 0.941	0.154	0.39
Red umbrella thorn	*β*_3_	− 0.823	0.148	0.439
Umbrella thorn	*β*_4_	− 3.692	0.523	0.025
Treatment thinned	*β*_5_	− 0.582	0.297	0.559
Post-thinning age	*β*_6_	0.107	0.036	1.113
Arboricide aftercare applied	*β*_7_	0.571	0.307	1.77
Black-thorn acacia: thinned	*β*_8_	0.826	0.208	2.285
Blade thorn: thinned	*β*_9_	− 0.067	0.220	0.936
Red umbrella thorn: thinned	*β*_10_	− 1.211	0.281	0.298
Umbrella thorn: thinned	*β*_11_	2.195	0.557	8.984
**Random effects**				
		SD		
Subplot	d_*kji*_	0.0001		
Plot	c_*kj*_	0.131		
Block	b_*k*_	0.182

**Table 3:** Expected number of tree/shrubs counted per subplot (area = 113.1 m^2^) in the thinned and non-thinned treatment areas estimated from the generalized linear mixed-effects model (GLMM) model coefficients.

Treatment/species	Model coefficients (β)	Sum (β)	Mean counts exp(sum(β)	± 95% confidence level
**Non-thinned**				
Sickle bush	*β*_0_ + *β*_5_(0)	1.777	5.912	1.224
Black-thorn acacia	*β*_0_ + *β*_1_(1) + *β*_5_(0) + *β*_8_(0)	0.635	1.887	1.692
Blade thorn	*β*_0_ + *β*_2_(1) + *β*_5_(0) + *β*9(0)	0.836	2.307	1.653
Red umbrella thorn	*β*_0_ + *β*_3_(1) + *β*_5_(0) + *β*_10_(0)	0.954	2.595	1.633
Umbrella thorn	*β*0 + *β*_4_(1) + *β*_5_(0) + *β*_11_(0)	− 1.915	0.147	3.401
**Thinned**				
Sickle bush	*β*_0_ + *β*_5_(1)	1.195	3.305	2.367
Black-thorn acacia	*β*_0_ + *β*_1_(1) + *β*_5_(1) + *β*_8_(1)	0.879	2.41	4.910
Blade thorn	*β*_0_ + *β*_2_(1) + *β*_5_(1) + *β*_9_(1)	0.188	1.207	4.914
Red umbrella thorn	*β*_0_ + *β*_3_(1) + *β*_5_(1) + *β*_10_(1)	− 0.839	0.432	5.468
Umbrella thorn	*β*_0_ + *β*_4_(1) + *β*_5_(1) + *β*_11_(1)	− 0.302	0.74	19.511

The parameter estimate *β*_*5*_ refers to the mean count of sickle bush specimens in the thinned treatment area (Tables 2 and 3). The number of sickle bush specimens was 55.9% of the expected amount in the non-thinned area (non-thinned = 5.91, thinned = 3.31). For the other species, less individuals were also recorded in the thinned area, with a difference of 47.7% for blade thorn and 83.4% for red umbrella thorn compared to the non-thinned area. In contrast, the mean counts in the thinned area were greater by 21.7% for black-thorn acacia and by 80.1% for umbrella thorn than amounts compared to the non-thinned area. The overall number of individuals counted in the thinned area was 8.09 (716 individuals ha^−1^) (Tables 2 and 3, Fig. 2).

Wald chi-square tests revealed statistically significant differences in the mean counts between tree/shrub types (Table 4). Differences in the mean tree/shrub counts between treatments was statistically significant (*P*-value < 0.001). The post-hoc tests of treatments by tree/shrub type showed significant differences between treatments for red umbrella thorn (*P*-value < 0.001) (negative response to the thinning treatment), and umbrella thorn (*P*-value = 0.039) (positive response to the thinning treatment). The response to thinning was also positive for black-thorn acacia, and negative for sickle bush and blade thorn, although statistically insignificant. Post-thinning age was significantly and positively related to the number of tree/shrub counts. Thus, mean tree/shrub count increased by 11% concomitant with every annual increase in post-thinning age. The effect of arboricide aftercare treatment was positive on mean tree/shrub count, but was statistically insignificant.

**Table 4:** Hypothesis tests of the estimated generalized linear mixed-effects model (GLMM) coefficients showing overall treatment effects and differences in the abundance of encroacher species in the thinned and non-thinned treatment areas.

Variable	χ^2^	df	Pr(> Chisq)
Tree/shrub types	114.137	4	0.000
Post-thinning age	8.798	1	0.003
Aftercare	3.477	1	0.062
**Effect of treatment by tree/shrub type**			
Overall	63.606	5	0.000
Sickle bush	3.862	1	0.247
Black-thorn acacia	0.575	1	1
Blade thorn	3.845	1	0.249
Red umbrella thorn	23.026	1	0.000
Umbrella thorn	7.068	1	0.039

*P*-values for the effect of treatment by tree/shrub type were adjusted for multiple testing using the Bonferroni method.

### Effect of treatment on the tree/shrub equivalent (TE) densities recommended for the area and approximate woody biomass availability

**Figure 3:**
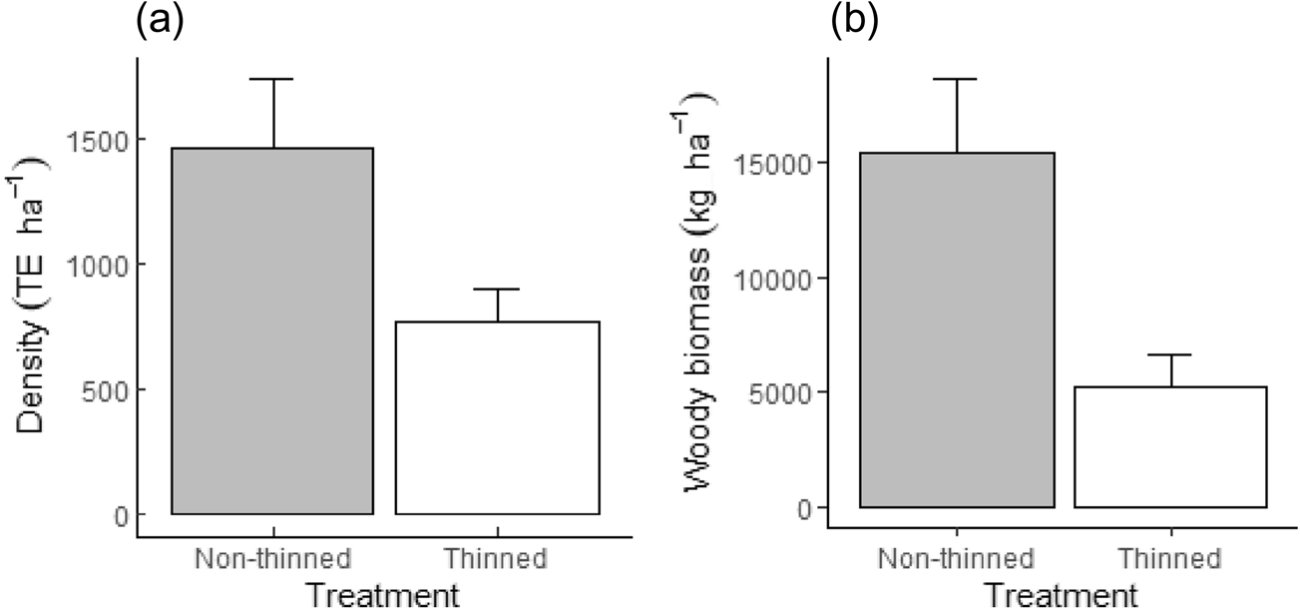
Expected (**a**) densities (TE ha^−1^), and (**b**) dry wood biomass (kg ha^−1^) of encroaching trees/shrubs in the thinned and non-thinned treatment areas estimated from the linear mixed-effects (LME) model coefficients. Error bars indicate ± 95% confidence level of the variability in tree/shrub densities (TE ha^−1^) and woody biomass (kg ha^−1^) of the non-thinned and thinned areas.

The overall densities (TE) for encroaching species measured in the non-thinned area (*β*_*0*_) was, on average, 1464.05 TE ha^−1^ (± 95% CI = 269.93) (Table 5a and Fig. 3a). The negative value for the effect of thinning treatment (*β*_*1*_) indicates that the overall mean was significantly less (by 693.09 TE ha^−1^; *P*-value = 0.001) than the baseline value (*β*_*0*_). This led to a mean estimate of 770.95 TE ha^−1^ (95% CI 128.49), which was 47.3% less than the amounts in the non-thinned area. Our results indicate that mean TE densities were 59.01% greater in the non-thinned area and 13.4% greater in the thinned area than the minimum recommended density for a 400 mm rainfall area (i.e., 600 TE ha^−1^).

**Table 5:** Linear mixed-effects (LME) models showing the effects of fixed factors (treatment, post-thinning age) on (a) tree/shrub densities expressed as TE ha^−1^, and (b) woody biomass of encroaching trees/shrubs.

	Parameter (β)	(a) Density (TE ha^−1^)			(b) Wood biomass (kg ha^−1^)		
		Estimate	SE	*P*-value	Estimate	SE	*P*-value
**Fixed effects**							
(Intercept)	*β*_*0*_	1464.05	137.1	< 0.001	15,387.73	1630.468	< 0.001
Treatment-thinned	*β*_*1*_	− 693.095	151.841	0.001	− 10,167.4	1785.401	< 0.001
Post-thinning age	*β*_*2*_				639.96	227.849	0.017
**Random effects**							
		SD			SD		
Plot	c_*kj*_	0.207			7.466		
Block	b_*k*_	0.004			3.847

Mean woody biomass of encroaching species in the non-thinned area (*β*_*0*_) was 15.4 tonnes ha^−1^ (± 95% CI 3.2) (Table 5b and Fig. 3b). The effect of thinning treatment (*β*_*1*_) resulted in a mean difference of 10.2 tonnes ha^−1^, which was significantly less (*P*-value < 0.001) than the biomass in the non-thinned treatment (*β*_*0*_). Hence, the overall woody biomass in the thinned area was, on average, 5.2 tonnes ha^−1^ (± 95% CI 1.4) and 66.1% less than the biomass in the non-thinned treatment. The effect of post-thinning age had a significant (*P*-value = 0.017) and positive trend on the mean woody biomass estimates, which corresponded to a 0.64 tonne ha^−1^ change in biomass concomitant with an annual increase in post-thinning age.

### Effect of treatment on the vegetation structure

#### Stem sizes

**Figure 4:**
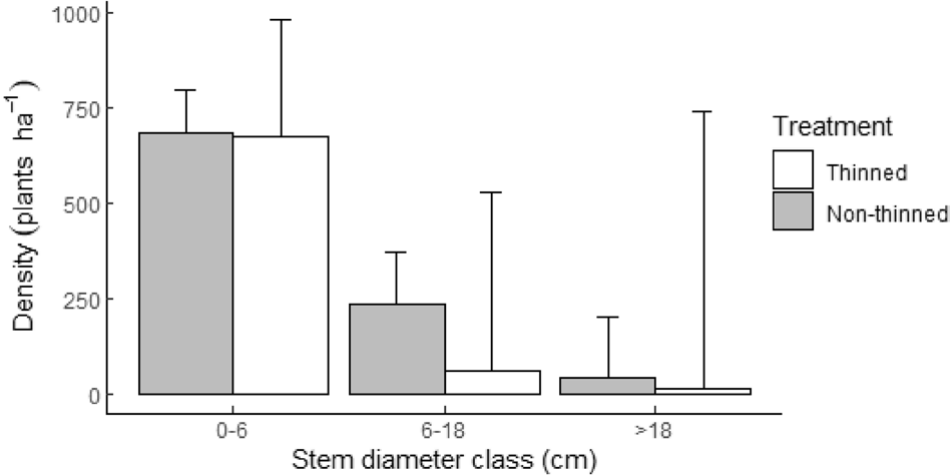
Expected tree/shrub density per hectare in the non-thinned and thinned treatment areas estimated from the generalized linear mixed-effects model (GLMM) model coefficients in stem classes, 0–6 cm, 6–18 cm and ≥ 18 cm. Error bars indicate ± 95% confidence level of the variability in tree/shrub densities of the three measured stem classes of the non-thinned and thinned areas.

The number of trees/shrubs in stem diameter class ≤ 6 cm in the non-thinned area (*β*_0_) was, on average, 7.74 plants per subplot (684.12 plants ha^−1^) (Tables 6 and 7, Fig. 4). The negative values for estimates in the other stem diameter classes showed that counts were less abundant than in the ≤ 6 cm diameter class: mean counts for the 6–18 cm and ≥ 18 cm classes differed by 65.2% and 93.4% times, respectively. The parameter estimate (*β*3) refers to the mean counts of trees/shrubs in stem diameter class ≤ 6 cm in the thinned treatment. The low value of parameter (*β*3) can be directly interpreted as the average relative change (1.2%) from the counts in the ≤ 6 cm diameter size class in the non-thinned area (non-thinned = 7.74, thinned = 7.65) (Tables 6 and 7). In comparison to the non-thinned area, mean counts in the thinned area were 74% and 68.3% lower for the 6–18 cm and ≥ 18 cm diameter classes, respectively (Tables 6 and 7, Fig. 4). Overall, our predictions showed that the majority (79.1%) of the measured stem diameters were in the ≤ 6 cm diameter class.

**Table 6:** Generalized linear mixed-effects model (GLMM) showing the effects of fixed factors (treatment, post-thinning age, and arboricide aftercare) on the abundance of encroaching trees/shrubs measured in stem classes, 0–6, 6–18 and ≥ 18 cm.

Variable	Parameter (β)	Value	SE	exp (β)
**Fixed part**				
(Intercept)	*β*_0_	2.046	0.133	7.737
6–18 cm	*β*_1_	− 1.057	0.078	0.348
≥ 18 cm	*β*_2_	− 2.723	0.160	0.066
Treatment thinned	*β*_3_	− 0.012	0.441	0.988
Post-thinning age	*β*_4_	0.087	0.055	1.091
Arboricide aftercare applied	*β*_5_	0.250	0.494	1.284
6–18 cm: thinned	*β*_6_	− 1.336	0.140	0.263
≥ 18 cm: thinned	*β*_7_	− 1.138	0.284	0.321
**Random part**				
		SD		
Subplot	d_*kji*_	0.316		
Plot	c_*kj*_	0.315		
Block	b_*k*_	0.309

**Table 7:** Expected tree/shrub density per subplot (area: 113.1 m^2^) in the non-thinned and thinned treatment areas estimated from the generalized linear mixed-effects model (GLMM) coefficients in stem classes, 0–6 cm., 6–18 cm. and ≥ 18 cm.

Treatment/stem diameter class	Model coefficients (β)	Sum (β)	Mean counts (exp(sum(β))	± 95% confidence level
**Non-thinned**				
≤ 6 cm	*β*_0_ + *β*_3_(0)	2.046	7.737	1.297
6 − 18 cm	*β*_0_ + *β*_1_(1) + *β*_3_(0) + *β*_6_(0)	0.989	2.69	1.512
> 18 cm	*β*_0_ + *β*_2_(1) + *β*_3_(0) + *β*_7_(0)	− 0.677	0.508	1.775
**Thinned**				
≤ 6 cm	*β*_0_ + *β*_3_(1)	2.034	7.648	3.454
6–18 cm	*β*_0_ + *β*_1_(1) + *β*_3_(1) + *β*_6_(1)	− 0.358	0.699	5.297
> 18 cm	*β*_0_ + *β*_2_(1) + *β*_3_(1) + *β*_7_ (1)	− 1.827	0.161	8.239

Wald chi-square tests revealed statistically significant differences in the average counts among the different stem classes (*P*-value < 0.001) (Table 8). Differences in the mean tree/shrub counts per stem size between treatments were significant (*P*-value < 0.001); the post-hoc tests of treatment by stem diameter size showed significant differences between treatments in the 6–18 cm stem diameter class (*P*-value = 0.009), with a negative response to the thinning treatment. A negative response to thinning was also observed in the ≤ 6 cm and ≥ 18 cm stem diameter classes, but were statistically insignificant. Both post-thinning age and aftercare did not have any significant effect on the number of tree/shrubs present in each stem diameter class.

**Table 8:** Hypothesis tests of the estimated generalized linear mixed-effects model (GLMM) model coefficients showing overall treatment effects and differences in the abundance of encroacher species in stem classes 0–6 cm, 6–18 cm and ≥ 18 cm.

Variable	χ^2^	Df	*P*-value
Stem diameter class	422.357	2	< 0.001
Post-thinning age	2.511	1	0.113
Aftercare	0.258	1	0.611
**Effects of treatment by stem diameter class**			
Overall	103.17	3	< 0.001
≤ 6 cm	0.001	1	1
6–18 cm	8.762	1	0.009
≥ 18 cm	4.934	1	0.079

*P*-values for the effect of treatment by tree/shrub counts in the different stem classes were adjusted for multiple testing using the Bonferroni method.

#### Vegetation structure (vertical, horizontal) and habitat sighting lines

**Figure 5:**
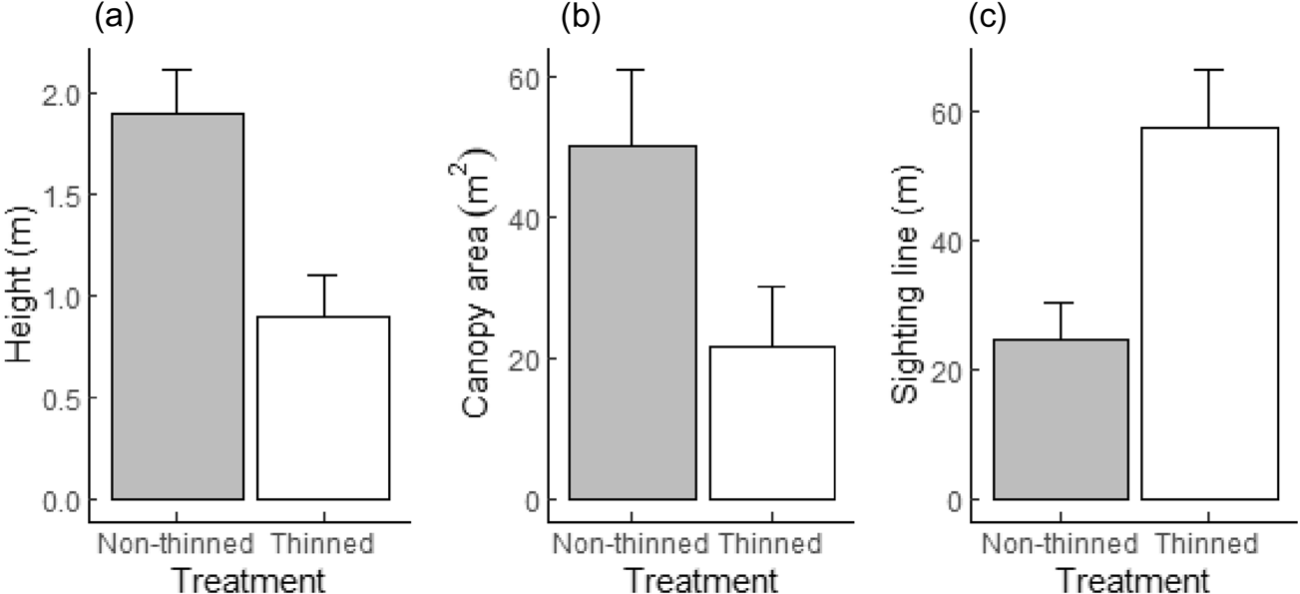
Expected mean (**a**) height (m), and (**b**) canopy area (m^2^) of bush-encroaching trees/shrubs, as well as (**c**) sighting lines (m) in the thinned and non-thinned treatment areas estimated from the linear mixed effects (LME) model coefficients. Error bars indicate ± 95% confidence level of the variability in tree/shrub height, canopy area and sighting lines of the non-thinned and thinned areas.

The mean height of trees/shrubs in the non-thinned area (*β*_*0*_) was 1.9 m (± 95% CI 2.1) (Table 9a and Fig. 5a). The effect of thinning treatment (*β*_*1*_) resulted in a significant (*P*-value < 0.001) reduction in height (1 m) compared to the non-thinned area (*β*_*0*_). Thus, for the thinned area a mean height of 0.9 m (± 95% CI 0.21) was predicted.

**Table 9:** Linear mixed effects (LME) models showing the effects of fixed factors (treatment, post-thinning age) on the vegetation structure (height, canopy area) of encroaching trees/shrubs, as well as on sighting lines in the thinned and non-thinned treatment areas.

	Parameter (β)	(a) Heights			(b) Canopy area			(c) Sighting lines		
		Estimate	Std. error	*P*-value	Estimate	Std. error	*P*-value	Estimate	Std. error	*P*-value
**Fixed effects**										
(Intercept)	*β*_*0*_	1.9	0.108	< 0.001	50.266	5.339	< 0.001	24.773	2.776	< 0.001
Treatment-thinned	*β*_*1*_	− 0.996	0.15	< 0.001	− 28.642	6.914	0.001	32.618	3.733	< 0.001
Post-thinning age	*β*_*2*_							− 4.937	0.902	< 0.001
**Random effects**										
		SD			SD			SD		
Subplot	d_*kji*_	0.427								
Plot	c_*kj*_	0.347			14.546			0.014		
Block	b_*k*_	0.001			0.014			9.015

The overall canopy area measured per subplot (area: 113.1m^2^) in the non-thinned area (*β*_*0*_) was, on average, 50.27 m^2^ (± 95% CI 10.51) (Table 9b and Fig. 5b). Thinning treatment (*β*_*1*_) resulted in a significant (*P*-value = 0.001) reduction in canopy area (28.64 m^2^) in relation to the non-thinned (*β*_*0*_) area. As such, mean canopy area in the thinned area was 21.62 m^2^ (± 95% CI 8.65) per subplot, which was equivalent to a 57% reduction in woody canopy cover of encroaching species compared to the non-thinned area. Correspondingly, the woody canopy cover per subplot in the non-thinned area equates to 44.4% (± 95% CI 9.30); for the thinned area 19.1% (± 95% CI 7.65). The estimates showed greater random variability in the average tree/shrub heights and canopy areas amongst the plots than between blocks (plot pairs).

For habitat sighting lines, a total of 1173 measurements were taken in 293 subplots, of which 50.6% were in the thinned and 49.5% were in the non-thinned areas. The mean sighting line measured in the non-thinned area was 24.8 m (± 95% CI 8.73 m) (Table 9c and Fig. 5c). The effect of thinning treatment (*β*_*1*_) resulted in a greater mean sighting line of 57.4 m (± 95% CI 5.47 m): a statistically significant (*P*-value < 0.001) difference of 32.62 m. Post-thinning age (*β*_2_) had a significant and negative effect on the sighting lines. Thus, mean sighting lines would decrease by 4.94 m concomitant with every annual increase in post-thinning age.

## Discussion

### Number of individuals and observed species-treatment interactions

Our results indicate that sickle bush had the greatest mean abundance, followed by black-thorn acacia, blade-thorn and red umbrella thorn. Non-thinned plots had the greatest abundance of trees/shrubs, and overall tree/shrub-treatment interactions were significant. In non-thinned areas, the abundance of the main encroacher species, sickle bush and black-thorn acacia (Tables 2 and 3) were lower than the average estimates for encroached habitat in this region^3,57^. We expect those regional estimates were influenced by factors that included rainfall, soil type, fire history, number of sample sites and management of the area, which could cause inaccuracies in extrapolations over larger areas. Our data provides detailed information regarding the extent of bush encroachment, as well as the effects of thinning at the localised level.

With regard to red umbrella thorn, a negative and significant response to thinning was observed; umbrella thorn had a positive and significant response to thinning. Contrary to our expectations, the most abundant species, sickle bush, was not reduced significantly by thinning, possibly due to the avoidance of small-sized stems^58^, and this may explain the substantial reduction in red umbrella thorn as it was structurally larger (Table 1). The significantly greater abundance of umbrella thorn in the thinned area shows that thinning dramatically accelerated regeneration of this species, possibly due to reduced competition and also because the retained trees/shrubs may have increased their reproductive output^33,59^. These results are consistent with those reported by Nghikembua et al., (2021) who found that the occurrence probabilities for red umbrella thorn were significantly less, and were greater for umbrella thorn in the same thinned plots.

The response to thinning was not significant for other species, which may be partly due to the arboricide aftercare application that prevented regeneration via coppicing. Also, as species abundance at the mean 7.2-year mark time since thinning were still poor, this may indicate that a longer regeneration period is required for these species. Inventories of bush control operations performed elsewhere have shown that in the absence of aftercare or timely aftercare applications, re-encroachment can occur within a short period (< 5 years) due to coppicing stumps and root suckering^2,21^. Moreover, in these areas, regardless of chemical aftercare, mechanically cleared sites showed greater levels of re-encroachment, possibly due to soil disturbance, delayed timing of the chemical aftercare, and greater probability of leaving stumps untreated.

A slight increase in black-thorn acacia counts by 21.7% was observed in the thinned plots. According to Joubert et al., (2013), significantly higher seed production and sapling establishment for this species were positively related with exceptional high rainfall seasons (at least two consecutive years). Also, survival rates of seedlings were five times greater away from trees (in open gaps) than under trees. It is likely that recruitment from seed occurred in our study area during 2008– 2012 when exceptional, above average, annual rainfall (> 444 mm) was recorded^45^.

### Tree/shrub equivalent densities (TE) recommended for the area and approximate woody biomass availability

The mean overall TE density for the non-thinned area showed a moderate level of encroachment and exceeded twice the recommended threshold for the area. The estimated density was lower than in other areas of the country where ~ 6,000 TE ha^−1^ has been observed^60^. Also, overall woody biomass (Table 5b) in the non-thinned areas was much less than the regional average (36.2 tonnes ha^−1^)^21^. However, the estimates in our study correspond closely with previous studies conducted on the same farms^61,62^. Possible explanations for reduced woody biomass volumes include differences in species composition, soil type, climatic factors, management history, as well as methodologies applied in biomass estimation^35,36,39^. The sickle bush specimens that were dominant in our study area were structurally smaller (Table 1), which would also affect the total woody biomass value. Thinning treatment was effective in causing a significant reduction in tree/shrub densities, well within the recommended levels (600–750 TE ha^−1^) for the area. Correspondingly, woody biomass was significantly reduced. The mean difference of 10.2 tonnes ha^−1^ dry woody biomass between treatments suggests that this amount may have been removed during the thinning operations. Based on these estimates, we expect that our study area of 17,422.44 ha contains ~ 268,091.8 tonnes (equates to 0.268 Mt) of woody biomass. The Namibian Ministry of Environment, Forestry and Tourism (MEFT) Guidelines highlight the importance of avoiding complete clearance of trees/shrubs by retaining a minimum density consistent with the rainfall received in the area^2^. During the thinning operations, harvesters are trained to identify target and protected species, to select harvestable size classes, and to leave small clumps of undisturbed vegetation to provide browsing and cover for biodiversity.

### Effect of treatment on vegetation structure and sighting lines

Thinning was effective in modifying the vegetation structure as shown by the significant differences between the treatments in vegetation structure and the abundance of small-medium sized trees/shrubs (6–18 cm stem class). It was evident that following thinning, the overall mean height, canopy size and abundance of trees/shrubs were significantly less than in the non-thinned areas. Moreover, thinned areas exhibited longer sighting lines, which were significantly different to the non-thinned areas (Table 9). The obvious explanation for these results is the considerable reduction in the overall tree/shrub abundance (Tables 2 and 3) in the thinned plots, with the number of individuals in the 1–3 m and > 3 m height classes significantly less than in the non-thinned areas^45^.

The mean sighting line (57.4 m) in the thinned areas in this study is less than those measured in sparsely vegetated areas preferred by cheetahs reported in other studies^4,52^ in the same region. The shorter mean sightline in the thinned areas in our study could be due partly to the fact that thinning was carried out conservatively in this area with the aim to retain specific tree/shrub densities rather than the densities that would result from natural disturbances (e.g. fire, natural dieback) or when bush removal was carried out for different management objectives (e.g. clearing for a grass fields). Also, our predictions show that sighting lines would decline with progressing post-thinning age and may take ~ 14 years to equalise between treatments. One possible explanation for this occurrence is the rapid natural regeneration present in the same study area due to re-encroachment by young cohorts (0–1 m height class)^45^. Another possible explanation for reduced sighting lines is the accelerated growth of structural features (e.g. canopy, stems) due to the absence of competition amongst the retained vegetation^10,33,59,63,64^.

A reduction in tree/shrub densities would be advantageous as it would restore the soil moisture balance, reduce the suppression effects of woody plants on the herbaceous layer, thereby increasing the likelihood for greater grazing capacity and ease of movement as animals manoeuvre through the habitat^1,3,4,12,21,65–67^. This may explain why thinned areas were strongly frequented by wildlife in the same plots in another study^28^.

## Conclusion and management recommendations

Our results indicate that thinning was effective in reducing the overall abundance of trees/shrubs, which was still evident at the average 7.2-year mark since thinning and when arboricide aftercare was applied. Of the measured trees/shrubs, red umbrella thorn was significantly reduced, and umbrella thorn significantly increased in the thinned areas. This suggests that thinning has the potential to change the floristic composition of species within an ecosystem, as preferred species are reduced (e.g. red umbrella thorn) and low-density species are promoted (e.g. umbrella thorn). The effect of thinning treatment was not significant for sickle bush, blade thorn and black-thorn acacia. However, the difference in expected counts for these species may indicate the true thinning treatment effect, which is just too small to be significant with our sample size.

This study has revealed that trees/shrubs can be selectively harvested, well within the recommended densities (600–750 TE ha^−1^) for the area. The average difference in woody biomass between the treatments (10.4 tonnes ha^−1^) suggests that this amount could be harvested within the recommended tree/shrub density range for the area. Thinning also modified the vegetation structure; tall, dense shrubland with limited sighting lines in the non-thinned areas was restored to short and low-cover shrubland with greater sighting lines. We anticipate that a low woody canopy cover and tree/shrub abundance would restore the soil moisture balance, promote perennial grass cover, and increase the grazing capacity. Also, the sparsely vegetated thinned areas would be an ideal habitat for wildlife that rely on longer sighting lines when hunting or evading predators.

This study revealed important findings regarding the effects of treatment on the encroaching woody vegetation and success of restoration thinning. However, it is not without any limitations. Firstly, there is uncertainty as to how climate change induced weather patterns (e.g., sporadic rainfall, increased drought frequencies) could influence the regeneration rate of woody species and grass cover, which could determine the restoration success and its duration. For example, enhanced plant growth, seed production and recruitment of samplings are expected during years of exceptional rainfall^68^. Over the long term, and without post thinning management, this could further promote bush encroachment, reduce sighting lines and grazing capacity. Equally, during drought years, poor plant growth, less grass cover and more overgrazing are expected and could further promote woody seedling establishment in subsequent above average rainfall years due to the loss of the herbaceous layer^3^. The uncertainties of these weather patterns demand better adaptive rangeland management strategies consistent with climate change including regulating livestock stocking rates, rotation grazing, and post thinning management of previously thinned areas. Long term monitoring into the patterns of the vegetation structure in relation to treatment (thinned, non-thinned) is necessary, particularly in response to climate change induced weather patterns.

Secondly, the results were limited to the study period, number of plots observed and a single thinning methodology; therefore, longer-term data, replicating the study over larger areas (multiple farms) and stratifying different levels of thinning are required to fully evaluate the effects on the vegetation structure. For future research and assessment, we recommend: (1) long term monitoring of regrowth to reveal trends and rates of regeneration by species, (2) thinning of established saplings in restored areas to prevent loss of longer sighting lines and re-encroachment, (3) development of allometric equations to predict available biomass, and (4) ongoing restoration to control bush encroachment to increase rangeland productivity and suitable habitats for species that prefer an open savannah vegetation structure.

## Supplementary Information

Supplementary Information.

## Data Availability

The datasets used in deriving the results during the current study are available from the corresponding author on reasonable request.
