# Supplementary material for: Response of woody vegetation to bush thinning on freehold farmlands in north-central Namibia
Source: Sci Rep. 2023 Jan 6;13:297. doi: 10.1038/s41598-022-26639-4 (PMC9822995; doi:10.1038/s41598-022-26639-4)
Supplement: Supplementary file 1 — Supplementary Information. [file 41598_2022_26639_MOESM1_ESM.pdf]

Appendix A

Regression model equations (power: $y = ax^b$) for calculating aboveground woody biomass. Abbreviations; HT= height, SD = stem diameter: where y = woody biomass; a = intercept, x = independent variable/s (tree/shrub height, stem diameter or combination) and b = slope.

Species	Independent Variable	Biomass Stems/branches	Model	n	r	r^2
Black-thorn Acacia	HT	>2cm (wood)	$y = 135.53x^{3.8701}$	15	0.91	0.83
	HT & SD	>2cm(wood)	$y = 44047x^{1.5456}$	15	0.92	0.85
	HT	≤2cm (bush feed)	$y = 1270.6x^{2.0933}$	15	0.91	0.83
	HT & SD	≤2cm (bush feed)	$y = 31469x^{0.8608}$	15	0.93	0.87
Red umbrella thorn	HT	>2cm (wood)	$y = 143.01x^{3.3147}$	15	0.93	0.86
	HT & SD	>2cm (wood)	$y = 91930x^{1.7774}$	15	0.96	0.93
	HT	≤2cm (bush feed)	$y = 816.25x^{2.1754}$	15	0.87	0.76
	HT & SD	≤2cm (bush feed)	$y = 39394x^{1.005}$	15	0.92	0.84
Microphyllous	HT	>2cm (wood)	$y = 143.35x^{3.6046}$	15	0.91	0.83
	HT & SD	>2cm (wood)	$y = 62601x^{1.6463}$	15	0.93	0.87
	HT	≤2cm (bush feed)	$y = 1492.4x^{1.7591}$	15	0.77	0.60
	HT & SD	≤2cm (bush feed)	$y = 31791x^{0.8467}$	15	0.83	0.69
